# Jasmonates in the Ethylene-Induced Resistance of Detached Citrus Fruits to Peel Damage

**DOI:** 10.3390/ijms26104805

**Published:** 2025-05-17

**Authors:** María T. Lafuente, Raúl Sampedro, Paco Romero

**Affiliations:** Postharvest Physiology and Biotechnology for Food Sustainability Laboratory, Department of Food Biotechnology, Instituto de Agroquímica y Tecnología de Alimentos (IATA-CSIC), Av. Agustín Escardino 7, 46980 Paterna, Valencia, Spain; rsampedro@iata.csic.es (R.S.); promero@iata.csic.es (P.R.)

**Keywords:** carbon starvation, jasmonate, phytohormone interaction, quality loss, transcriptional regulation

## Abstract

It is known that nutrient deprivation following detachment can cause non-chilling peel pitting (NCPP) in citrus fruits when stored under a non-stressful environment and that this damage is reduced by pretreating the fruit with ethylene (ETH) (4 d, 10 µL L^−1^). The present work investigates the effect of this pretreatment on jasmonate (JA) accumulation and transcriptional regulation in mature Navelate oranges (*Citrus sinensis* L. Osbeck) stored under non-stressful conditions. ETH increased the expression of abundant genes participating in the synthesis of *cis*-(+)-12-oxo-phytodienoic acid (OPDA), jasmonic acid (JA), and methyl jasmonate (MeJA). ETH also upregulated genes involved in jasmonoyl–isoleucine (JAIle) synthesis (*CsJAR1*) and decrease (*CsCYP94B3* and *CYP94C1*), and *CsSTA2*, related to JA sulfation. The levels of these JA metabolites increased during fruit holding in ETH and after shifting them to air, with MeJA accumulation being especially remarkable. Overall, the beneficial effect of ETH on reducing NCPP appears to be related not only to this redirection of OPDA and JA metabolism towards the formation of JA derivatives but also to the regulation of JA signalling. Indeed, the repression of the receptor *CsCOI1* and upregulation of various *CsJAZs* repressors caused by nutrient deprivation, together with the ETH-mediated induction of *CsCOI1*, *CsTOPLESS*, and abundant *CsJAZs* during long-term storage, suggests the occurrence of an ETH-enhanced negative transcriptional regulatory feedback loop in JA metabolism and signalling, by which the susceptibility of detached Navelate oranges to NCPP might be reduced.

## 1. Introduction

Hormones are involved in the regulation of the quality, ripening, and senescence of various horticultural crops and play important roles in their adaptation to stresses that lead to deterioration in their appearance and postharvest losses [1,2,3]. Many citrus fruit cultivars are prone to developing postharvest non-chilling peel pitting (NCPP), also known as rindstaining. This physiological disorder manifests as depressed peel areas that may increase in size and number during postharvest storage (Supplemental Figure S1). NCPP does not alter the internal quality of citrus fruits, but it causes the depreciation of their external quality, thereby leading to important food waste and economic losses. This disorder can be either produced by environmental conditions favouring sudden changes in the water status of citrus peel tissues [4,5] or by carbon starvation that occurs as a consequence of fruit detachment [6,7]. Previous reports showed that conditioning fully coloured mature oranges with high ethylene (ETH) levels (10 µL L^−1^ for 4 d) reduces NCPP when the fruits are stored under non-stressful environmental conditions [7]. This ETH pretreatment causes sub-lethal stress, as it induces plant stress-related responses, at the same time that it reduces NCPP, which indicates that it favours cross protection against the subsequent postharvest carbon shortage stress in citrus fruit [7]. The rise in ETH production found during that treatment [7] was also a good indicator of stress, as mature citrus fruits exhibit autoinhibitory ETH production under non-stressful conditions [8] and an increase in this hormone when exposed to stresses [7,9] or an inhibition of ETH perception [10].

The importance of jasmonates (JAs) as signalling molecules in plants’ responses to stresses is well known [11,12]. The application of JA-related compounds has a positive effect on the quality and postharvest storage of fruit [2,13] and may reduce postharvest physiological disorders manifested as peel pitting and/or necrosis in different fruit [14,15,16,17,18]. ETH and JA metabolism and signalling in plants and fruits may act independently, antagonistically, or jointly against stresses or in the regulation of physiological processes affecting the quality and postharvest behaviour of the products [19,20,21,22,23]. Other reports have shown the relevance of JA metabolism and signalling in the elicitation of resistance against biotic and abiotic stresses causing postharvest losses in citrus fruit [3], and more recently, it has been shown that fruit degreening with an ETH releasing compound (ethephon) at a dose that barely modifies the citrus fruit ethylene production varies the concentration of some JA-related metabolites [24]. However, transcriptional events associated with degreening and such metabolic changes have not been investigated. Likewise, it is still unknown whether there is a link between the ETH treatment and JA metabolism and signalling in the peel of fully coloured mature citrus fruit and whether this link is relevant to the efficacy of the ETH pretreatment on reducing NCPP. In this regard, it must be mentioned that the mechanisms induced by ETH in citrus fruit differ with the fruit’s physiological stage and the ETH dose applied [7,25].

The role of JAs in protecting citrus fruit from the deleterious consequences of nutrient deprivation caused by the detachment of this sink organ has also not been reported in spite of the importance of this stress, which is enhanced during postharvest conditions, as fruit respiration increases together with the carbon demand to sustain it. As far as we know, there is only one report linking carbon shortage and JA metabolism in fruits (kiwifruit), but it was performed in attached fruit [26]. It is also worth mentioning that treating harvested citrus fruit with ATP as an external energy source reduces NCPP, induces a transient increase in ETH production [6], and alters the expression of genes participating in lipid β-oxidation [6], which is an important step in the JA biosynthesis pathway [11]. To the best of our knowledge, there is scarce information linking JA perception and signalling and carbon starvation in plants [27,28], although no reports have focused on detached fruit. It is also worth noting that JAs may participate in early responses to stresses by acting not only as signalling messengers but also in long-term responses associated with the containment of cell damage propagation [3,11,15,29].

Considering the relevance of JAs to protecting plants and fruits against diverse stresses leading to damage and the existing interplay between ETH and JAs, our working hypothesis is that JA metabolism and/or signalling participate in the ETH-induced cross-adaptation against NCPP of citrus fruit and that they are involved in the response to nutrient deprivation stress driven by detachment. For this reason, we have also examined the effect of nutrient deprivation stress on the regulation of JA metabolic pathways under non-stressful environmental postharvest conditions (20 °C and 90–95% relative humidity (RH)). On the one hand, a comprehensive transcriptional analysis was performed by selecting 25 genes involved in JA metabolism and signalling. And on the other, changes in the levels of the jasmonic acid (JA) precursor *cis*-(+)-12-oxo-phytodienoic acid (OPDA), which may mediate plant responses to stresses by itself [30,31,32], of JA, and of the JA derivatives methyl jasmonate (MeJA) and jasmonoyl–isoleucine (JAIle), were also determined.

## 2. Results

In order to understand the effect of ETH and nutrient deprivation driven by detachment on the transcriptional regulation of the JA biosynthesis and signalling pathways [11,33], a set of 25 genes that participate in these processes were studied in the present work.

### 2.1. ETH Pretreatment of Detached Citrus Fruits and Postharvest Nutrient Deprivation Stress Modify the Regulation of Genes Involved in OPDA Synthesis

**Figure 1:**
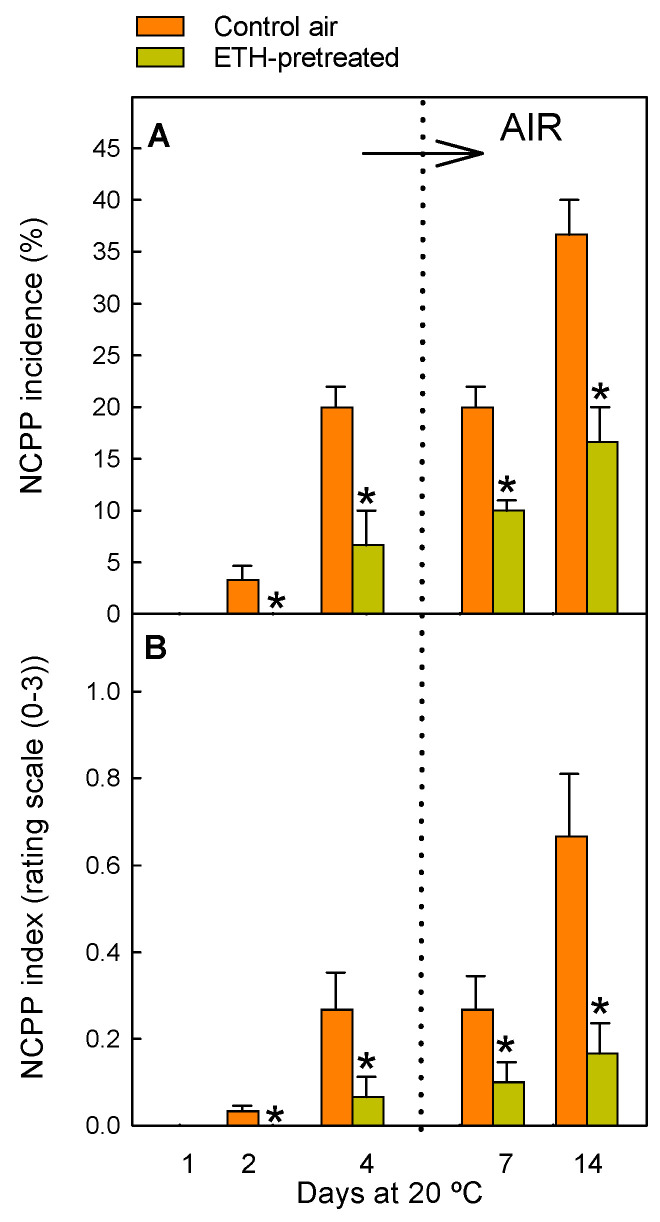
Changes in NCPP incidence (**A**) and severity (**B**) in Navelate oranges pretreated with 10 µL L^−1^ ETH at 20 °C and 90–95% RH for 4 d and then stored in air at the same temperature and RH (orange bars), and in the control oranges stored for up to 14 d in air under the same environmental conditions (yellow bars). The horizontal arrow labelled ‘AIR’ and the vertical dotted line in the plots indicate the transfer of the ETH-conditioned fruit to air, whereas the control fruits were constantly maintained in air. Data shown correspond to the means of three biological replicates, and the standard error is indicated by the error interval. Significant differences (*p* < 0.05) between the oranges pretreated and not (control) with ETH for the same storage day are indicated by the asterisks.

In order to understand the putative role of JAs on the beneficial effect of fruit ETH conditioning on reducing NCPP damage in fully coloured citrus fruits, a treatment of 10 µL L^−1^ ETH for 4 d was applied to detached Navelate oranges. As expected, the results confirmed the efficacy of the ETH pretreatment on reducing the incidence (Figure 1A) and severity (Figure 1B) of this disorder in the fruits stored in a non-stressful environment (non-chilling temperature and saturated RH). By day 4, the % of fruit showing NCPP was reduced by about three-fold in the oranges held in ETH, with respect to the control fruit maintained in air, and such a percentage was still two-fold lower in the ETH-pretreated fruit when they were transferred for 10 additional days to air (14 d) (Figure 1A). Likewise, the NCPP severity was reduced four-fold by the ETH pretreatment at day 14 (Figure 1B). Once the efficacy of the treatment on reducing NCPP was confirmed, its effect on JA metabolism was investigated.

**Figure 2:**
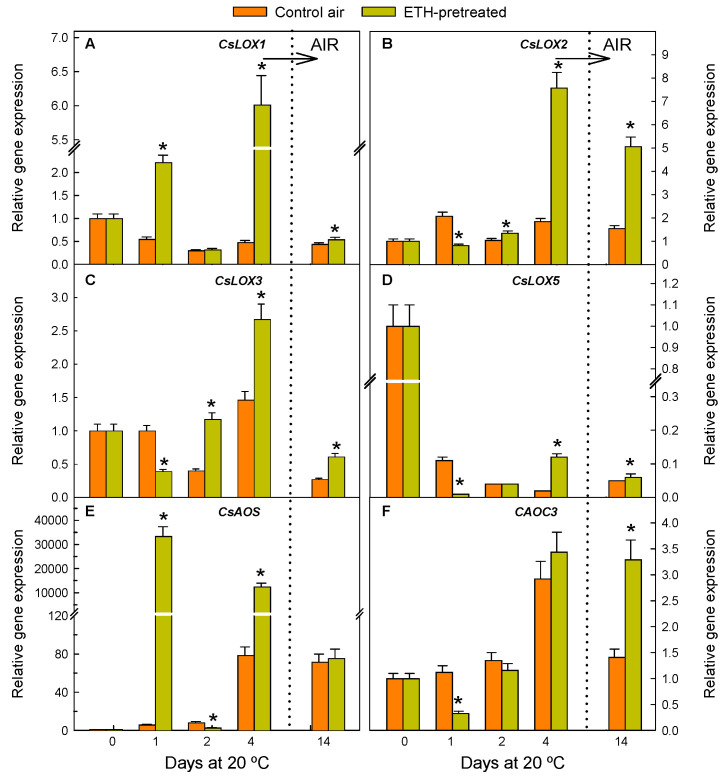
Changes in the relative expression levels of the genes involved in the synthesis of OPDA in the flavedo of fruit pretreated with 10 µL L^−1^ ETH at 20 °C and 90–95% RH for 4 d and then stored in air at the same temperature and RH (orange bars), and in the control oranges stored for up to 14 d in air under the same environmental conditions (yellow bars). The horizontal arrow labelled ‘AIR’ and the vertical dotted line in the plots indicate the transfer of the ETH-conditioned fruit to air, whereas the control fruits were constantly held in air. Data shown correspond to the means of three biological replicates, and the standard error is indicated by the error interval. Significant differences (*p* < 0.05) between the oranges pretreated and not (control) with ETH for the same storage day are indicated by the asterisks. (**A**) *CsLOX1*; (**B**) *CsLOX2*; (**C**) *CsLOX3*; (**D**) *CsLOX5*; (**E**) *CsAOS*; and (**F**) *CsAOC3*.

Changes in the expression of genes involved in the synthesis of the JA precursor OPDA, which takes place in the chloroplast and encodes lipoxygenase (LOX), allene oxide synthase (AOS), and allene oxide cyclase (AOC) proteins, are shown in Figure 2. Three patterns of changes in the expression of these genes were observed during fruit holding under the ETH atmosphere (0–4 d, Figure 2). First, a transient and sharp ETH-induced accumulation of *CsLOX1* (Figure 2A) and *CsAOS* (Figure 2E) was observed at the beginning of ETH exposure (1 d), when NCPP was not yet visible in any sample (Figure 1), and at the end of the treatment (4 d). In this regard, the great effect of ETH on increasing *CsAOS* (5700-fold by 1 d, 175-fold by 4 d), as compared to the other JA-related genes (Figure 2), is remarkable.

On the other hand, the expression of *CsLOX2* (Figure 2B), *CsLOX3* (Figure 2C), and *CsLOX5* (Figure 2D) increased with respect to the control fruit after long-term ETH exposure (4 d), when the efficacy of the ETH treatment on reducing NCPP was already evident, although the accumulation of these transcripts decreased in response to ETH treatment by 1 d. Lastly, the *CsAOC3* gene expression was also transiently downregulated by 1 d, but no significant differences were found between the ETH-treated fruit and the control fruit at any other period during the hormone treatment (2–4 d, Figure 2F). After fruit transference from ETH to air for 10 additional days (14 d), the transcript levels of all the genes participating in OPDA biosynthesis remained unchanged, except for *CsLOX3* and *CsAOC3*, which were significantly reduced (Figure 2). Nevertheless, the expression of all these genes but *CsAOS* was higher in the ETH-pretreated fruit than in the control fruit by this period (Figure 2).

It is also worth noticing that detaching Navelate fruits, and the subsequent stress caused by nutrient deprivation, induced, per se (control fruit), a sharp drop in the expression of *CsLOX5*, and of *CsLOX1* to a minor extent, by day 1 (Figure 2D), and an upregulation of *CsAOS* and *CsAOC3* by 4 d (Figure 2E,F). Detachment had a fluctuating effect on the expression of *CsLOX2* and *CsLOX3* (Figure 2B,C). The expression levels of *CsLOX1*, *CsLOX2*, *CsLOX5*, and *CsAOS* remained nearly constant from 4 to 14 d, but those of *CsLOX3* and *CsAOC3* sharply decreased during this storage period (Figure 2).

### 2.2. ETH Pretreatment and Nutrient Deprivation Modify the Postharvest Regulation of Genes Involved in JA, MeJA, and JAIle Accumulation

**Figure 3:**
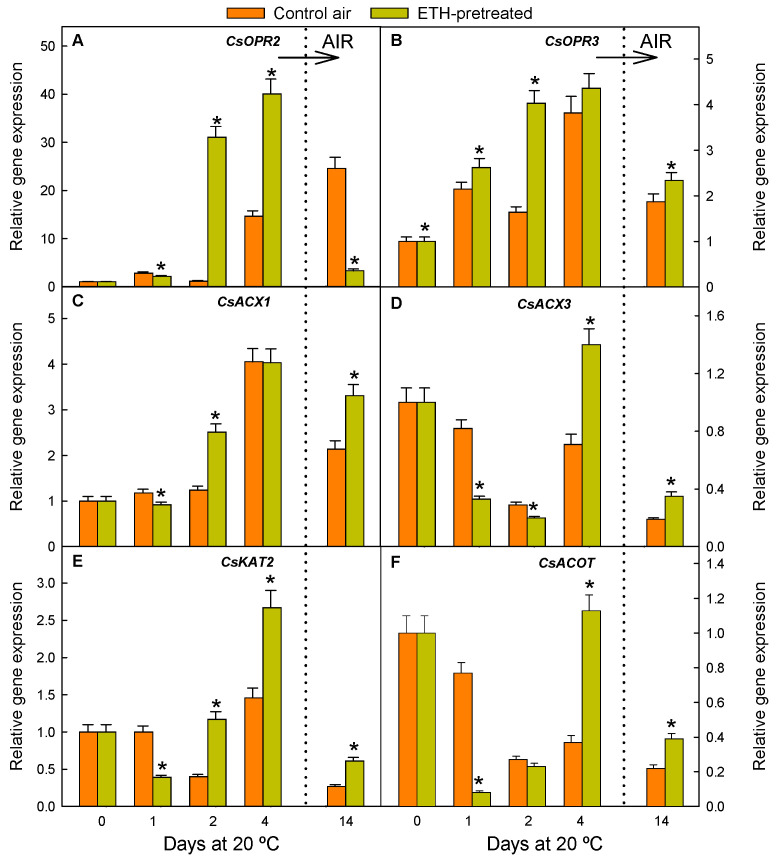
Changes in the relative expression levels of the genes involved in the synthesis of JA from OPDA in the flavedo of the fruit pretreated with 10 µL L^−1^ ETH at 20 °C and 90–95% RH for 4 d and then stored in air at the same temperature and RH (orange bars), and in the control oranges stored for up to 14 d under the same environmental conditions (yellow bars). The horizontal arrow labelled ‘AIR’ and the vertical dotted line in the plots indicate the transfer of the ETH-conditioned fruit to air, whereas the control fruits were constantly held in air. Data shown correspond to the means of three biological replicates, and the standard error is indicated by the error interval. Significant differences (*p* < 0.05) between the oranges pretreated and not (control) with ETH for the same storage day are indicated by the asterisks. (**A**) *CsOPR2*; (**B**) *CsOPR3*; (**C**) *CsACX1*; (**D**) *CsACX3*; (**E**) *CsKAT2*; and (**F**) *CsACOT*.

To understand the metabolisation of OPDA into JA, which occurs after OPDA translocation to peroxisomes, changes in the expression of two gene isoforms encoding the 12-oxo-phytodienoic acid reductases (OPR), the genes participating in the β-oxidation of fatty acids, encoding two acyl-coenzyme A oxidase isoforms (ACX) and an L-3-ketoacyl CoA thiolase (KAT), and an acyl-CoA thioesterase (ACOT)-encoding gene, were determined (Figure 3).

All the genes participating in JA synthesis, except for *CsOPR3*, were downregulated after short-term ETH fruit exposure (1 d), while a marked effect of ETH on upregulating all the genes was observed at longer periods (2 and/or 4 d) (Figure 3). The relative expression of *CsOPR3*, *CsACX1*, *CsACX3*, *CsKAT2*, and *CsACOT* markedly decreased after shifting the fruit from ETH to air (14 d) (Figure 3). Similarly, the transcript levels of all the genes but *CsOPR2* were still higher in the ETH-pretreated than in the control fruit by day 14 (Figure 3).

**Figure 4:**
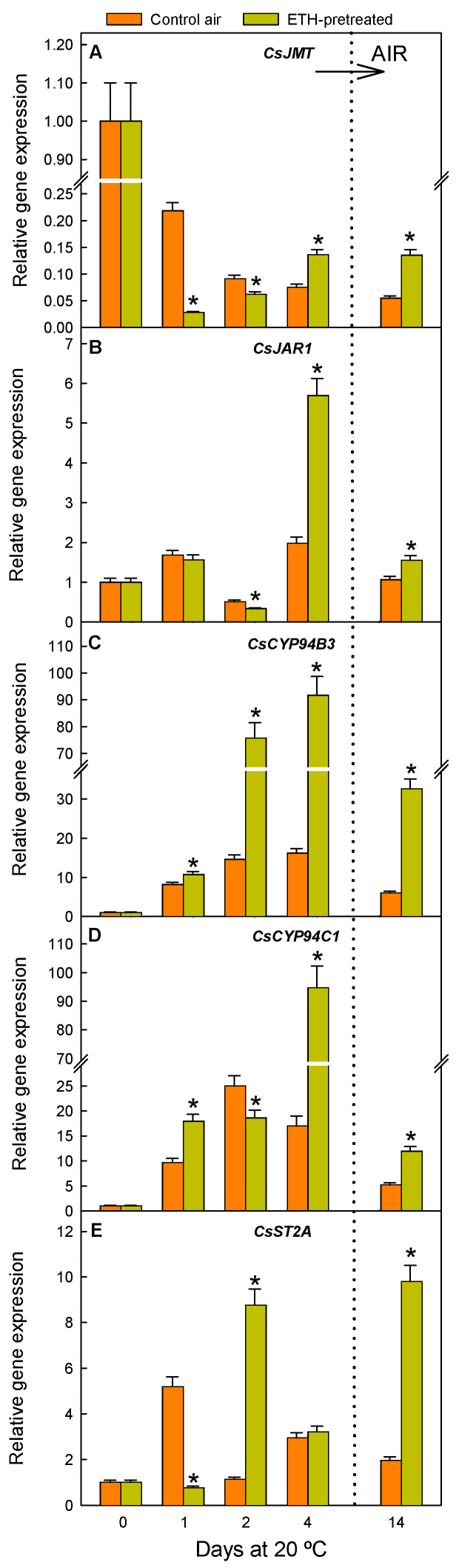
Changes in the relative expression levels of the genes involved in the accumulation of MeJA (*CsJMT*) (**A**), in JAIle synthesis (*CsJAR1*) (**B**), in catabolism (*CsCYP94B3* and *CsCYP94C1*) (**C**,**D**), and in the formation of the JAIle derivative 12-hydroxy jasmonyl sulfate (*CsST2A*) (**E**) in the flavedo of fruit pretreated with 10 µL L^−1^ ETH at 20 °C and 90–95% RH for 4 d and then stored in air at the same temperature and RH (orange bars), and in the control oranges stored for up to 14 d in air under the same environmental conditions (yellow bars). The horizontal arrow labelled ‘AIR’ and the vertical dotted line in the plots indicate the transfer of the ETH-conditioned fruit to air, whereas the control fruits were constantly held in air. Data shown correspond to the means of three biological replicates, and the standard error is indicated by the error interval. Significant differences (*p* < 0.05) between the oranges pretreated and not (control) with ETH for the same storage day are indicated by the asterisks.

Regarding MeJA and JAIle accumulation, which takes place after JA transport to the cytosol, we examined the transcriptional changes of a JA carboxyl methyltransferase (JMT) (Figure 4A), involved in MeJA accumulation, and a jasmonoyl–isoleucine synthetase 1 (JAR1) (Figure 4B), which conjugates JA with isoleucine to yield JAIle. Moreover, we determined changes in gene expression of a sulfotransferase ST2A participating in other JA derivatives (JA-S) (Figure 4E) and of genes encoding two P450 cytochromes (CYP94B3 and CYP94C1) (Figure 4C,D) participating in JAIle catabolism before JAIle diffuses into the nucleus to bind the SCF^COI1^ receptor complex [11,33].

The expression of *CsJMT* was lower in the ETH-pretreated than in the control non-treated fruit by 1 and 2 d (Figure 4A). After long-term ETH exposure (4 d), the *CsJMT* expression was already higher in the ETH-pretreated fruit, and this effect remained when the fruits were shifted to air (14 d). Regarding the genes related to JAIle metabolism, the results indicated that ETH had an important impact on upregulating *CsJAR1* (Figure 4B), as well as the two cytochrome-encoding genes (*CsCYP94B3* and *CsCYP94C1*) related to its degradation, with this effect being especially noticeable by 2 and 4 d (Figure 4C,D). The ETH treatment also induced a transient increase in the expression of the *CsST2A* sulfotransferase-encoding gene (Figure 4E) by day 2. After long-term storage upon fruit transference to air, the expression of all the JAIle-related genes sharply increased in the ETH-pretreated as compared to the control fruits.

As observed in the fruits that were always maintained in air, fruit nutrient deprivation caused by detachment induced relevant changes in the regulation of genes involved in JA, MeJA, and JAIle metabolism (Figure 4), and many changes occurred immediately after fruit harvest. The expression of *CsOPR2* sharply increased by day 4 and kept increasing until 14 d in the control fruit stored in air (Figure 3A). The *CsOPR3* expression increased by 1 d and reached a maximum by 4 d (Figure 3B), while that of *CsACX1* (Figure 3C) remained nearly constant initially and then sharply increased by day 4. In contrast, the expression of *CsACX3* (Figure 3D), *CsKAT2* (Figure 3E), and *CsACOT* (Figure 3F) decreased by 1–2 d to transiently increase by day 4. The fact that the relative expression of *CsACX3* and *CsACOT* was always lower than in the freshly harvested fruit (day 0) merits attention (Figure 3D,F). Contrary to that observed for *CsOPR2*, the relative expression of the other genes participating in the JA synthesis decreased from 4 to 14 d (Figure 3); and, by day 14, it was especially very low for *CsACX3* (Figure 3D), *CsKAT2* (Figure 3E), and *CsACOT* (Figure 3F). On the other hand, the expression of *CsJMT*, related to MeJA synthesis, continuously decreased as a consequence of detachment in the fruits held in air for up to 14 d (Figure 4A), while that of genes positively or negatively affecting JAIle accumulation increased immediately after fruit detachment (Figure 4B–E). Such increases were transient and especially great for *CsCYP94B3* and *CsCYP94C1* (Figure 4C,D), whose expressions increased 12 and 24 times, respectively, 2 d after detachment. Changes in *CsJAR1* and *CsST2A* (Figure 4B,E) transcript levels fluctuated similarly throughout the storage period but were less relevant as compared to the other genes. Still, *CsST2A* increased by 5.5-fold immediately after fruit harvest (1 d).

### 2.3. ETH Pretreatment and Nutrient Deprivation Modify the JA Levels in Detached Citrus Fruit

**Figure 5:**
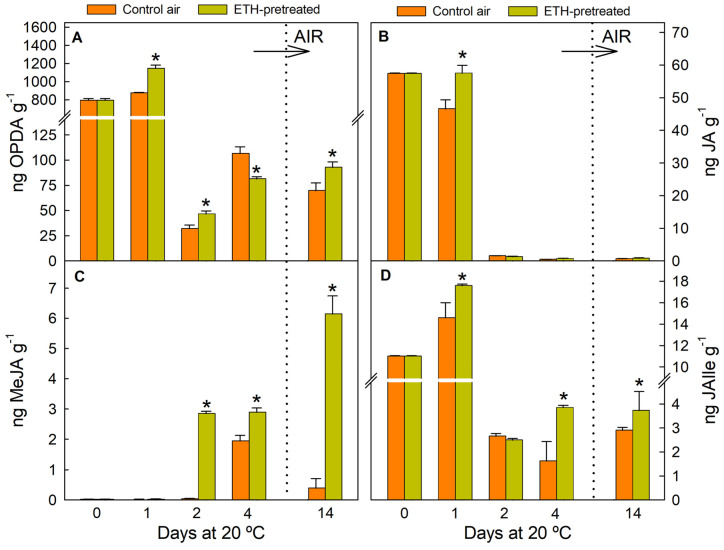
Changes in the accumulation of OPDA (**A**), JA (**B**), MeJA (**C**), and JAILe (**D**) in the flavedo of fruit pretreated with 10 µL L^−1^ ETH at 20 °C and 90–95% RH for 4 d and then stored in air at the same temperature and RH (orange bars), and in the control oranges stored for up to 14 d under the same environmental conditions (yellow bars). The horizontal arrow labelled ‘AIR’ and the vertical dotted line in the plots indicate the transfer of the ETH-conditioned fruit to air, whereas the control fruits were constantly held in air. Data shown correspond to the means of three biological replicates, and the standard error is indicated by the error interval. Significant differences (*p* < 0.05) between the oranges pretreated and not (control) with ETH for the same storage day are indicated by the asterisks.

The marked effect of the ETH pretreatment on increasing MeJA, with respect to the other JA-related metabolites (Figure 5), is worth noticing. Moreover, MeJA further increased upon fruit transference from ETH to air (Figure 5C), and by 14 d, it was about 12-fold higher in the ETH-treated fruit than in the control fruit. The ETH-induced increases in the levels of the other metabolites determined were lower than two-fold in any storage period (Figure 5). The treatment had little effect on increasing the OPDA concentration (Figure 5A), albeit the concentration of this metabolite was much higher than that of MeJA in the flavedo and did not avoid the sharp OPDA decline caused by fruit detachment in the control fruit. Similarly, ETH had little effect on the JA (Figure 5B) and JAIle (Figure 5D) concentrations, although JAIle levels were two-fold higher in the ETH-pretreated than in the control fruit by day 4. Regarding the stress caused by fruit detachment, it is remarkable that MeJA was almost undetectable until 4 d in the fruit continuously held in air (Figure 5C). In contrast, JA levels dropped by 2 d and were no longer detectable (Figure 5B), whereas JAIle slightly increased shortly after detachment (1 d) but sharply decreased thereafter (Figure 5D).

### 2.4. JA Perception and Signalling Is Affected by the ETH Pretreatment and Nutrient Deprivation in Citrus Fruit

**Figure 6:**
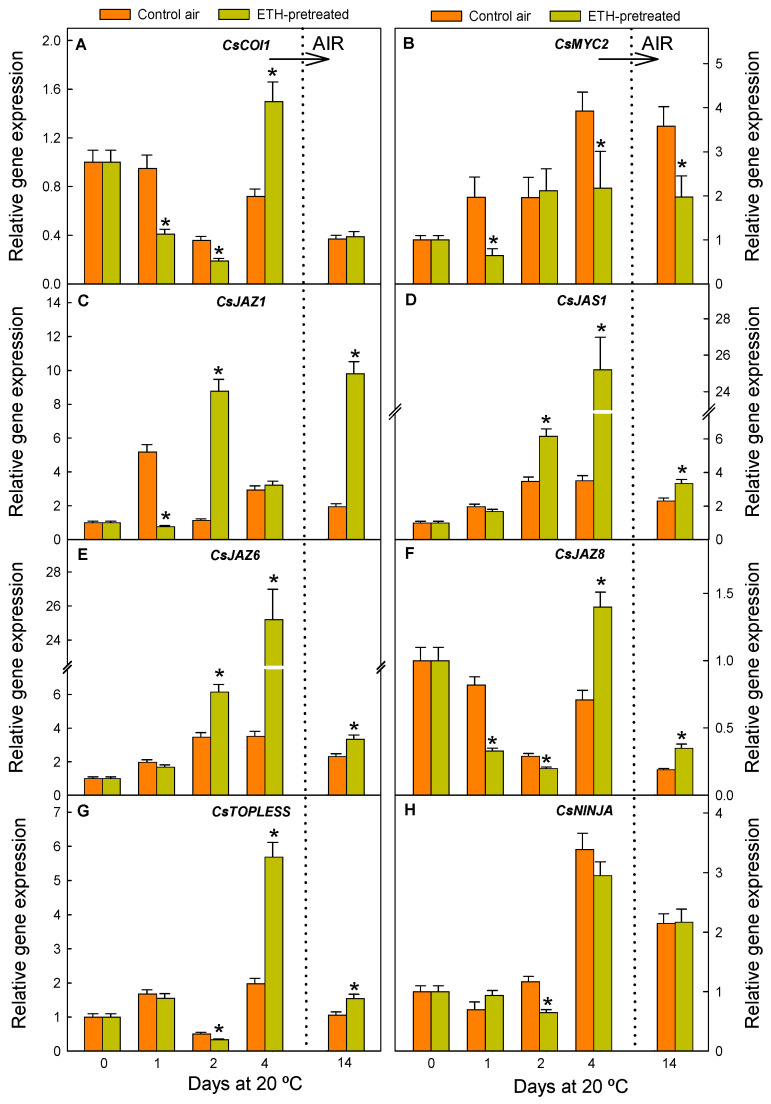
Changes in the relative expression levels of the genes involved in jasmonate perception and signalling in the flavedo of the fruit pretreated with 10 µL L^−1^ ETH at 20 °C and 90–95% RH for 4 d and then stored in air at the same temperature and RH (orange bars), and in the control oranges stored for up to 14 d in air under the same environmental conditions (yellow bars). The horizontal arrow labelled ‘AIR’ and the vertical dotted line in the plots indicate the transfer of the ETH-conditioned fruit to air, whereas the control fruits were constantly held in air. Data shown correspond to the means of three biological replicates, and the standard error is indicated by the error interval. Significant differences (*p* < 0.05) between the oranges pretreated and not (control) with ETH for the same storage day are indicated by the asterisks. (**A**) *CsCOI1*; (**B**) *CsMYC2*; (**C**) *CsJAZ1*; (**D**) *CsJAS1*; (**E**) *CsJAZ6*; (**F**) *Cs CsJAZ8;* (**G**) *CsTOPLESS;* and (**H**) *CsNINJA*.

Regarding JA perception and signalling, changes in the expression of several components of the SCF^COI1^ receptor complex, including the receptor coronatine insensitive 1 (COI1) (Figure 6A) and different jasmonate-zim domain (JAZ) repressors (Figure 6C–F), which block the response of the hormone, were determined. Likewise, changes in the transcript levels of genes coding for TOPLESS (Figure 6G) and the novel interactor of JAZ (NINJA) (Figure 6H) proteins were evaluated because of their contribution to the reduction in the pool of the transcriptional repression complex (JAZ-NINJA-TOPLESS). This complex should be repressed for inducing the JA-mediated protective responses against stresses [33]. Lastly, we examined changes in the expression of the *MYC2* transcription factor (TF) (Figure 6B), due to its relevance in favouring the activation of the downstream signal after the degradation of the JAZ proteins.

The ETH treatment modified JA signalling in citrus fruit (Figure 6). The expression of the JA receptor (*CsCOI1*) initially decreased (1–2 d) in response to ETH before NCPP was detected (Figure 1), but it noticeably increased by 4 d (Figure 6A), when NCPP was more severe in the control than in the ETH-treated fruit (Figure 1). *CsCOI1* expression decreased in the ETH-treated fruit shifted to air (14 d) until it reached similar transcript levels to its control fruits (Figure 6A). Overall, maintaining the fruit under ETH reduced the expression of the *CsMYC2* transcription factor by days 1 and 4 (Figure 6B). ETH also had a marked effect on inducing the expression of all negative regulators coding for JAZ or TOPLESS proteins by days 2 and/or 4 (Figure 6C–G), although *CsJAZ1* and *CsJAZ8* were markedly repressed by the hormone immediately after fruit detachment (1 d). Upon fruit transference to air (14 d), the expression of these negative regulators was still markedly greater in the ETH-treated fruit than in the control fruit, with this effect being markedly greater in *CsJAZ1*. The transcript levels of the *CsNINJA* negative regulator, however, were barely affected by ETH, and a slight repression caused by the hormone was observed only by day 2 (Figure 6H).

As for the effect of nutrient deprivation stress on the regulation of JA perception and signalling, *CsCOI1* transcript accumulation transiently decreased, reaching its minimum levels by days 2 and 14 (Figure 6A), while the *CsMYC2* positive effector continuously increased in the control fruit due to detachment until 14 d (Figure 6B). The expression of *CsJAZ1*, *CsJAS1*, and *CsJAZ6* (Figure 6C–E) and that of the *TOPLESS* (Figure 6G) repressors of the signalling pathway slightly increased after detachment in the fruit continuously stored in air or transiently decreased by day 1 or 2 (*CsJAZ8* and *CsTOPLESS*) (Figure 6F,G). Conversely, *CsNINJA* expression sharply increased by 4 d in response to the nutrient deprivation stress (Figure 6H).

## 3. Discussion

ETH had an important impact on the transcriptional regulation of the 25 genes involved in JA metabolism and signalling selected in this study. In addition, the ETH conditioning treatment had an overall effect on increasing OPDA, JA, MeJA, and JAIle accumulation in the flavedo of fully coloured, non-climacteric citrus fruit.

The treatment upregulated *CsAOS* and all the selected *CsLOX* isoforms (involved in OPDA formation) in the flavedo, which agrees with findings in different fruits or their by-products [34,35,36], but contrasts with findings in apple fruit [37,38]. Some major effects of the ETH treatment on these genes were observed, in general, after long-term ETH exposure (4 d), but the relative expression of some of them peaked after both short- (1 d) and long-term (4 d) ETH exposure. Therefore, and considering that the treatment reduced NCPP (Figure 1), the existence of two mechanisms that alleviate peel damage might be proposed. One occurs immediately after ETH application (1 d), related to the hormone per se, while the other one occurs in the long term (4 d), which might be the consequence of the stress caused by long fruit exposure to high ETH levels (4 d, 10 µL L^−1^ ETH). This fits with previous findings in mature oranges that indicated that this treatment causes sub-lethal stress involving reactive oxygen species (ROS)-related responses and mild membrane and cell degradation, which leads to stress cross-protection against NCPP [7]. In this regard, it is remarkable that LOX can trigger defence responses aimed at mitigating cell damage propagation [15,39] and that a connection between ETH production, the upregulation of *CsLOXs*, and the beneficial effect of a heat-conditioning treatment that caused sub-lethal stress and reduced peel injury in chilled citrus fruit has been previously reported [9,40]. ETH also upregulated all the genes leading to JA synthesis (Figure 3), which agrees with data in other fruits [41,42] and in mandarins with increased ethylene production because of heat stress [9,40]. The ETH-induced increases in the expression of *CsLOX1*, *CsLOX5*, and *CsAOS* (Figure 2A,D,E), and of *CsOPR2*, *CsOPR3*, and *CsAOC3* (Figure 3A,B,F) decreased upon fruit transference to air. However, we cannot rule out the participation of these genes in the ETH effect on reducing NCPP. Indeed, the ETH-mediated regulation of these genes was sufficient to increase the formation of OPDA and JA in the treated fruit as compared to those held in air, which would lead to the later regulation of downstream protective responses against NCPP. In spite of this effect, the treatment was not sufficient to avoid the drop observed in OPDA and JA early after fruit harvesting (Figure 5A,B). The situation is not simple, but the fact that *CsLOX5* and *CsAOC3* were among the less affected genes by ETH could be interpreted to mean that these genes function as rate-limiting steps in OPDA synthesis and the subsequent JA accumulation. On the other hand, the ETH-induced upregulation of *CsACXs* and *CsKAT2* involved in JA synthesis might disagree with JA levels because of the tendency of the metabolic flux towards the formation of JA derivatives downstream in the pathway. Another plausible interpretation for these apparent discrepancies would be a desynchrony between the transcriptional and translational cellular machineries. In fact, OPDA is synthesised in the chloroplast, whose structural integrity is altered by an excess of ETH in the peel of citrus fruit [24]. Within that context, the overall results suggest that nutrient deprivation caused by fruit detachment drives the reduction in the JA and OPDA pools in favour of the formation of JA derivatives and that this effect is enhanced by the ETH treatment. This idea is supported by the fact that ETH had a marked effect on favouring MeJA and JAIle synthesis and accumulation and on upregulating the gene encoding ST2A, which is relevant in reducing the pool of precursors of active forms of JAs [43], thus mediating JAs catabolism in stressed plants [44], and regulating plant hormonal signalling [45] (Figure 3A–E).

The ETH effect on JAIle content in the mature Navelate oranges (Figure 4D) was subtle but evident soon after detachment (1 d) and at the end of the ETH conditioning treatment (4 d), which contrasts with the marked effect of ethephon on increasing JAIle during degreening in Midknight Valencia oranges [24]. This difference could be due to the influence of the fruit maturity stage and/or the hormone concentration applied. Our results agree with the ETH effect on upregulating the gene responsible for its synthesis (*CsJAR1*) but also of those involved in its catabolism (*CsCYP94B3* and *CsCYP94C1*) (Figure 4). The upregulation of both cytochromes can lead to energy generation [46], so it could be a mechanism to cope with the extra energy required to sustain the ETH-induced rise in fruit respiration [7]. The results presented herein also agree with others showing that both ETH and MeJA increase in citrus fruit exposed to abiotic stress [3,9]. It is remarkable that ETH caused about a 70-fold rise in MeJA accumulation when this metabolite was almost undetectable in the fruit held in air (2 d in Figure 5C). This first ETH-induced rise in MeJA preceded that of *CsJMT* expression, but both events were induced by 4 d and after fruit shifting to air (Figure 4A and Figure 5C). Therefore, although other *CsJMT* isoforms might contribute to the first MeJA rise, we cannot rule out the contribution of the gene selected in this study to the higher MeJA content in the ETH-treated fruit after long-term storage. Considering the advance in its accumulation and the sharp increases detected, MeJA should be an important player in the ETH-induced protection against NCPP. In this regard, it is worth noting that MeJA promotes lignin synthesis and cell wall strengthening in fruits [47,48], which have been related to the beneficial effect of ETH on reducing NCPP [6]. Moreover, MeJA can reduce membrane lipid degradation by maintaining energy charge levels in fruit [49], which would delay NCPP development. Numerous studies have pointed out the involvement of MeJA in a transcriptional regulatory loop to modulate the expression of genes involved in JA metabolism and signalling [35,39,47,48,50,51,52,53,54], but this hypothesis remains unstudied in citrus fruit. In this regard, it should be noted that our results indicate that the ETH-induced rise in MeJA was concomitant with or preceded the upregulation of 23 out of the 25 selected genes. This result encourages future research to investigate MeJA participation in ETH-induced gene regulation and to evaluate whether MeJA application reduces NCPP, hence opening up new perspectives for its use in reducing this disorder, which affects numerous citrus cultivars worldwide.

This is also the first report showing how JA signalling is affected by ETH in citrus fruit (Figure 6). The ETH treatment might enhance JA perception, as it upregulated *CsCOI1* after a prolonged ETH exposure. However, it downregulated the positive regulator of JA signalling (*CsMYC2*), which is in concordance with findings in peach fruit [54]. In turn, ETH upregulated all but *CsNINJA* JA negative regulators, and their expression was still higher after shifting the fruit to air (Figure 6). This is not an easy scenario to explain, but we interpret that a negative-regulatory feedback loop could be occurring in JA signalling, by which the ETH-mediated accumulation of the JA derivatives would drive the attenuation of the signal transduction downstream in the responsive pathway in order to avoid putative deleterious effects caused by a sustained perception of JAs.

The results also reveal, for the first time, that nutrient deprivation stress caused by detachment (air-stored fruit) has a notable effect on the JAs pathway. The upregulation of *CsAOS* and *CsAOC3* (Figure 2E,F) and the early sharp drops in *CsLOX1* and *CsLOX5* expressions (Figure 2A,D), which were followed by a very marked drop in OPDA (Figure 5A), are remarkable. Therefore, both genes coding for LOX could be relevant players in the OPDA decrease caused by fruit detachment and hence by nutrient and energy shortage of this sink organ. This agrees with the fact that *LOX* was upregulated in pears after the addition of ATP, which increased the fruit’s energy charge [55]. The drastic JA decrease (Figure 5B) could be due to the decline of its precursor (OPDA, Figure 5A) or to the downregulation, soon after detachment, of the genes involved in the last steps of JA synthesis (*CsACX3*, *CsKAT2*, and *CsACOT*) (Figure 3D–F). In the climacteric apple fruit, the JA decline that occurs after harvesting was related to ripening progression [38]. However, citrus fruit is non-climacteric, and fully mature fruit were selected in our experimental design. Likewise, the decreases in OPDA and JA were found immediately after fruit harvesting, and, therefore, they would not be related to fruit senescence, whose symptoms differ from those of NCPP in citrus fruit. Hence, as the fruits were not stored under stressful environmental conditions, the above-mentioned changes may be likely related to nutrient deprivation and to the high susceptibility of Navelate oranges to NCPP.

The results obtained in the control detached fruit stored under non-stressful environmental conditions (20 °C and 90–95% RH) suggest that nutrient deprivation per se also redirects JA metabolism towards the synthesis of MeJA and JAIle and the formation of sulfated JA derivatives. The decline in JAIle by day 2 could be a consequence of the JA decrease but also of the higher increase in the expression of genes involved in its catabolism (*CsCYP94B3* and *CsCYP94C1*) (Figure 4C,D) than in its synthesis (*CsJAR1*) (Figure 4B). The upregulation of both cryptochromes (Figure 4C,D) might occur to compensate for the lack of energy supply from the plant, as they encode fatty-acid-metabolising enzymes able to provide extra energy in plants [46]. The MeJA levels transiently increased by 4 d in the air-stored fruit (Figure 5B) after JA decline (Figure 5C). It could be interpreted that the rise in MeJA is a defence response to cope with nutrient deprivation since the ETH treatment had an enhancing effect on increasing MeJA content, advancing the increase in this metabolite by day 2, and reducing the incidence of NCPP. Further support for this idea comes from the fact that ATP reduces starvation and NCPP in Navelate oranges and activates oxidative stress defence responses and the lignin biosynthetic process in the flavedo [6], as well as from data demonstrating that MeJA induces these same mechanisms in citrus fruit [18,56]. Regarding JA signalling, the transient downregulation of the JA receptor *CsCOI1* (Figure 6A) coinciding with the drops in OPDA, JA, and/or JAIle merits attention. The upregulation of the JA negative regulators, including most *JAZs* (Figure 6), which are hallmarks of carbon starvation in plants, is also remarkable [28]. The global results indicate, therefore, that the lack of carbon/energy supply to the flavedo negatively affects JA perception and signalling in Navelate oranges, which could contribute to the high susceptibility of this cultivar to postharvest NCPP. This idea is in agreement with the ETH-mediated upregulation of *CsCOI1* (Figure 6A) and the reduction in NCPP incidence after this hormonal treatment (Figure 1).

## 4. Materials and Methods

### 4.1. Fruit Material and Postharvest Treatments

Fully coloured mature Navelate sweet oranges (*Citrus sinensis* L. Osbeck) of uniform size and free of damage were harvested from adult trees in Castellón (Spain). They were then quickly transported to the laboratory and assigned to two different groups, which contained an equal number of fruits (105 per group). Fruits in the first group were treated for 4 d with 10 µL L^–1^ ETH in sealed containers and then transferred for 10 additional days to air, whereas those of the second group were continuously maintained in air for up to 14 d (control sample). Both treatments were performed in the dark in controlled humidity and temperature storage rooms at 90–95% RH and 20 °C to avoid environmental stress and in the presence of calcium hydroxide to avoid the accumulation of CO_2_. The fruits in each group were divided into two subgroups. The first subgroup contained 5 fruits per biological replicate and storage period and was used for flavedo (outer peel part) sampling to determine changes in JA levels and in the regulation of genes participating in JA metabolism, perception, and signalling. The flavedo was separated from the fruit using a razor blade at each sampling point, homogenised with a coffee mill in liquid nitrogen and kept at −80 °C for later analyses. Ten fruits per biological replicate were included in the second subgroup to estimate the evolution of the incidence and severity of NCPP. All determinations were performed in three biological replicates.

### 4.2. Evaluation of NCPP Damage

The incidence of damage was estimated by calculating the percentage of fruits that developed NCPP during fruit storage. Moreover, the severity of NCPP was determined by using a rating scale ranging from 0 to 3, which was established in accordance with the surface damage (Supplemental Figure S1). The average NCPP index was calculated by using the following formula: NCPP index = Σ (NCPP scale (0–3) × number of fruits in each class)/total number of fruits.

### 4.3. Analysis of Jasmonate-Related Metabolites

The concentrations of OPDA, JA, MeJA, and JAIle were determined in the three biological flavedo sample replicates by ultra-high-performance chromatography/mass spectrometry (UHPLC/MS) (ExionLC AD system, Framingham, MA, USA) after extracting the samples with 70% methanol containing 1% glacial acetic acid and using prostaglandin B1 as an internal standard [57], as reported in citrus fruit in detail by [3]. All the samples of the experiment were extracted on the same day using microcentrifuge tubes with screw caps, which provided a tight and secure seal to avoid losses/evaporation of the extractant or MeJA. The samples were always maintained in the closed vials protected from light at 4 °C. The extracts were not concentrated under N_2_ flow or dried under vacuum-heat conditions to avoid MeJA volatilisation. The chromatograph was connected to a refrigerated ExionLC AD Autosampler, and all chromatographic conditions used, including those of the triple quadrupole mass spectrometer Sciex Qtrap 6500 plus (ExionLC AD system, Framingham, MA, USA), were the same previously reported in [3]. The concentrations of all JA-related metabolites were calculated by using calibration standard curves generated by using commercial standards, and the Analyst^®^ Software 1.5.0 (SCIEX) was used to process the quantitative data [3]. All data shown represent the mean values of three replicate samples ± the standard error.

### 4.4. Isolation of Total RNA and Synthesis of cDNA

The total RNA isolation and the cDNA synthesis were performed from flavedo (1 g) as previously described by [40]. Briefly, the RNA was extracted with a preheated (65 °C) mixture of the extraction buffer (10 mL) reported in [40] and phenol (5 mL). A chloroform/isoamilic acid solution (24:1, *v*/*v*) (5 mL) was used to wash the extract, and after phase separation, 5 mL of phenol/chloroform/isoamilic acid (25:24:1, *v*/*v*/*v*) was used to re-extract the aqueous phase [40]. The nucleic acids were precipitated with cold ethanol. The resulting pellets were processed as described in [40], and after precipitation with 12 M lithium chloride at 4 °C overnight, the precipitates were dissolved in sterile water (50 µL) after being washed with sodium acetate (3 M, pH 6.0). Genomic DNA contamination was removed by using Ribonuclease-free DNAse (Thermo Fisher Scientific, Wilmington, DE, USA) according to the method provided by the manufacturer. The concentration and integrity of total RNA were determined as described in [40]. The Maxima H Minus First Strand cDNA Synthesis Kit from Thermo Fisher Scientific (Wilmington, DE, USA) was used for the first-strand cDNA synthesis from the total RNA (2 μg) according to the instructions of the manufacturer.

### 4.5. Gene-Specific Primer Pairs and RT-qPCR Analysis

A set of 25 genes was selected to characterise the transcriptional regulation of JA biosynthesis and signalling pathways. The gene-specific primer pairs related to JA metabolism, perception, and signalling were designed by employing the Primer3Plus software (https://www.primer3plus.com/index.html (accessed on 12 March 2025)). The primers for the genes involved in OPDA synthesis (*LOX1*, *LOX2*, *LOX3*, *LOX5*, *AOS*, *AOC3*), in the metabolisation of OPDA into JA (*OPR2*, *OPR3*) and others participating in the β-oxidation of fatty acids (*ACX1*, *ACX3*, *KAT2*, *ACOT*) are listed in Supplemental Table S1. Likewise, primer pairs were also designed for genes involved in MeJA and JAIle accumulation (*JMT*, *JAR1*), as well as for the generation of other JA derivatives (*CYP94B3*, *CYP94C1*, *ST2A*). As for JA perception and signalling, primer pairs of genes of the receptor complex (*COI1*) for different negative regulators (*JAZ1*, *JAS1*, *JAZ6*, *JAZ8*, *NINJA*, *TOPLESS*) and for a positive effector of the downstream signal (*MYC2*) were designed. For most of these steps in the JA metabolism and signalling, different multigene families are involved. Therefore, the above-mentioned genes were selected considering previous information obtained by our group through transcriptomic analyses that indicated their involvement in the responses of citrus to different stresses [6,7,40]. Data normalisation was performed by using the *CsACT* and *CsTUB* genes. The sequences of their primers are also included in Supplemental Table S1. The amplification of cDNA and the determination of gene expression were performed as reported in [40] by using SYBR Green 1 Master and a LightCycler480 System from Roche Diagnostic and the ‘Relative Expression Software Tool v1.1’ (REST, https://www.gene-quantification.de/rest-paper.html (accessed on 5 March 2025)). The cDNA amplification was checked for at least 35 cycles (10 s at 95 °C, 5 s at 60 °C, and 10 s at 72 °C), and to determine the amplicon specificity, a melting curve analysis was conducted. Data related to gene expression were reported as relative gene expression, which was determined by referring the gene expression levels of each sample to those of the flavedo of freshly harvested fruit. Data shown for each sample are the mean value of three biological replicates and two technical replicates for each biological replicate.

### 4.6. Statistical Analysis

The values shown are the means of the three flavedo biological replicates of each sample ± standard error. Significant differences (*p* < 0.05) for the same storage period between the fruit pretreated and non-pretreated (control) with ETH were determined by a *t*-test performed employing the software Statgraphics Plus 4.0 from Manugistics, Inc. (Rockville, MD, USA).

## 5. Conclusions

The ETH treatment drives complex molecular mechanisms associated with JA metabolism and signalling that vary over time exposure in the peel of citrus fruit. The situation is not simple, and the effects of ETH on JA regulation are not straightforward. Indeed, some changes in gene expression transiently occur right after the treatment and indicate early responses to the hormone, while major inductions appear at long-term exposure and might be in part related to an ETH excess. In turn, several genes are notably modulated in response to the hormone at both the beginning and the end of the treatment. The marked transcriptional effects of ETH on the genes participating in the synthesis of OPDA, JA, MeJA, and JAIle do not completely align with the accumulation profiles of these metabolites over time, which makes this scenario even more challenging. This apparent discrepancy might be due to the induction of genes involved in both the synthesis and catabolism of a specific metabolite, as is the case for JA and other JA derivatives, but also to a desynchrony between the transcriptional process and the translation cellular machinery. Within this context, it should also be considered that some of these genes might be acting as rate-limiting steps for the synthesis or catabolism of a particular metabolite, hence affecting the other ones downstream in the pathway. Future studies should also take into account the possible occurrence of a negative-regulatory feedback loop between the ETH-mediated accumulation of JA derivatives and the latter attenuation of the signal transduction response to alleviate a sustained perception of JAs, which appears to be a very plausible explanation for the inconsistencies between the transcriptional and metabolic data. Still, the overall results indicate that the beneficial effect of ETH on reducing NCPP is more likely related to the marked increase in MeJA, and to JAIle to a lower extent, than to the accumulation of OPDA or JA. This is supported by the fact that the ETH pretreatment does not avoid the decline of these metabolites upon nutrient deprivation caused by fruit harvest, which appears to be related to the redirection of JA and its precursor towards the synthesis of MeJA, JAIle, and jasmonyl sulfate derivatives.

## Data Availability

The data presented in this study are available on request from the corresponding author.

## Supplementary Materials

The following supporting information can be downloaded at https://www.mdpi.com/article/10.3390/ijms26104805/s1.

## Abbreviations

The following abbreviations are used in this manuscript:

ACOT	Acyl-CoA thioesterase
ACX	Acyl-coenzyme A oxidase
AOC	Allene oxide cyclase
AOS	Allene oxide synthase
COI1	Coronatine insensitive 1
CYP94B3	Cytochrome P450 94B3
CYP94C1	Cytochrome P450 94C1
ETH	Ethylene
JAIle	Jasmonoyl–isoleucine
JA	Jasmonic acid
JAR1	Jasmonoyl–isoleucine synthetase 1
JAs	Jasmonates
JAZ	Jasmonate-zim domain
JMT	Jasmonic acid carboxyl methyltransferase
KAT	L-3-ketoacyl CoA thiolase
LOX	Lipoxygenase
MeJA	Methyl jasmonate
NCPP	Non-chilling peel pitting
NINJA	Novel interactor of JAZ
OPDA	*cis*-(+)-12-oxo-phytodienoic acid
OPR	12-oxo-phytodienoic acid reductase
ST2A	Sulfotransferase 2A
