# Supplementary material for: Jasmonates in the Ethylene-Induced Resistance of Detached Citrus Fruits to Peel Damage
Source: Int J Mol Sci. 2025 May 17;26(10):4805. doi: 10.3390/ijms26104805 (PMC12112142; doi:10.3390/ijms26104805)
Supplement: Supplementary file 1 [file ijms-26-04805-s001.zip › ijms-3581979-Supplementary Matetrial.pdf]

Supplemental Figure S1. NCPP symptoms and rating scale used to determine NCPP index

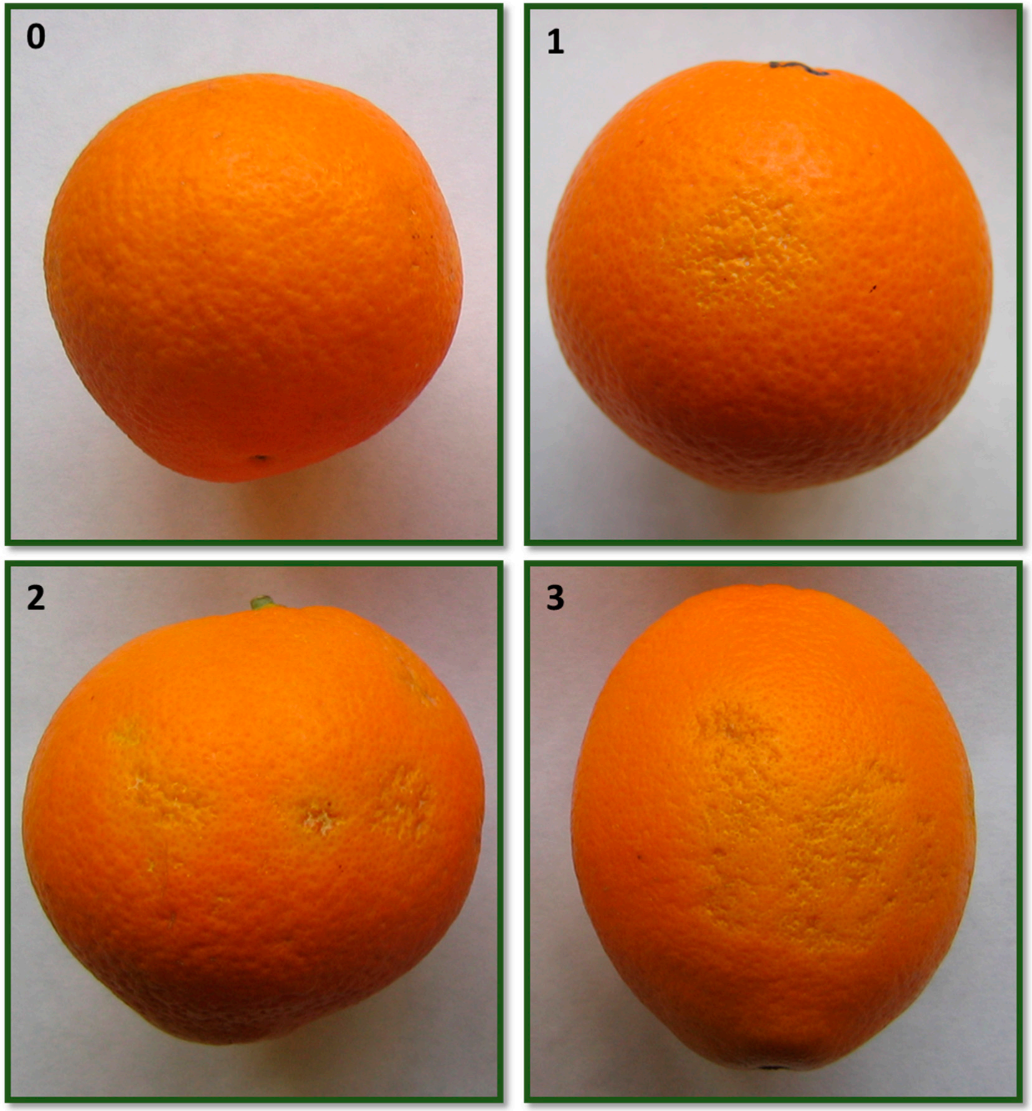

Supplemental Table S1. Primers used for RT-qPCR analyses

Citrus ID	Gene	Forward (5' - 3')	Reverse (5' - 3')
orange1.1g002839m.g	*CsLOX1*	CACGGCCTTCGTTTACTGAT	TGCACTGACTCTGATATGTT
orange1.1g002776m.g	*CsLOX5*	AACCATGGTGGCCTGAGATG	CTCAGCATACTCCGGGGTTC
orange1.1g043892m.g	*CSAOS*	GATTTGTGGGGGAGGGTGAG	TGATCCCGTCAACAGCTTCC
orange1.1g025155m.g	*CsAOC3*	CTTCAACAAACCCAGCAGCC	CCGGTGTAGAGCTTGTTCGT
orange1.1g017733m.g	*CsOPR2*	AGCAACTGGGGTTTCCAACA	GGGCGTCACTCCCTTAGAAG
orange1.1g025135m.g	*CsOPR3*	GCTTTGGCAGAAGATGGTGC	GTGATAGTGTTGCCTGGCCT
orange1.1g007504m.g	*CsACX1*	CGAGGCAGTTGCTTTATGGC	GCCAGCAATGGGAAGAGTCT
orange1.1g011790m.g	*CsACX3*	CGGGACTGGCTGTTCAATCT	ATTCCGGTGACCTCAAGCAG
orange1.1g012522m.g	*CsKAT2*	TCGGGACTGTCTGCTTCCTA	TGTGACACGCTTCTCCACTC
orange1.1g040957m.g	*CsACOT*	GGAAGAGCTACGCGAGAGAC	CCCTTTGGCTCTGAACCAGT
orange1.1g018119m.g	*CsJMT*	AATTCTTGTGGTGTTGGGAAGTAAC	CAGGGATTGCTGAGTGAAGCT
orange1.1g007464m.g	*CsJAR1*	ATGCGAAGTACCTGTCTGGAATC	CCCCTGCATAGTGCCTCAAC
orange1.1g001190m.g	*CsCYP94B3*	GCTTGTAGATGATGATGTTG	CGGAGGGTAAAGCCTCATGG
orange1.1g040199m.g	*CsCYP94C1*	TGAACTTCCGGACAACTGCA	GGATTTGGGGTCCGGATTGT
orange1.1g019118m.g	*CsST2A*	CCCCTGCTTACTTCCAATCCT	TTGGTTATTTGCATAGAGCTTGTATTC
orange1.1g007586m.g	*CsCOI1*	AGTTTTCGCAGGTTAGGGCA	GCCGCTTGACAATGGAGTTC
orange1.1g005651m.g	*CsMYC2*	GCCTGAGTCCGGGGAGATAT	CCCTCTCGAAGTAGGAGATC
orange1.1g028982m.g	*CsJAZ1*	ACATTTCTGCGGAGAAGGCA	CAGCAGGAGCCACTGATGAA
orange1.1g030695m.g	*CsJAS1*	CCTCGCCAAAGAAGCTTTCG	AGGAGCTGTTTCCGAAACGT
orange1.1g030183m.g	*CsJAZ6*	CAACCCCACTTCAGTTCCCA	TTGACCTTCAAGCTCCAGGC
orange1.1g046141m.g	*CsJAZ8*	TGAAGATTTCAGCCTCGCCG	GGTGATGCCGGTTCCATTTC
orange1.1g001190m.g	*CsTOPLESS*	GATTGGATTAACAGATGGAT	GCTTGTAGATGATGATGTTG
orange1.1g010436m.g	*CsNINJA*	TCAACTTCAGACTCCGCCAC	TCTGCCGAGATTGAGGAGGA
orange1.1g037845m.g	*CsACT*	TTAACCCCAAGGCCAACAGA	TCCCTCATAGATTGGTACAGTATGAGA
orange1.1g036688m.g	*CsTUB*	GCATCTTGAACCCGGTAC	ATCAATTCGGCGCCTTCAG
